# Supplementary figures and images for: Symmetry-enforced topological nodal planes at the Fermi surface of a chiral magnet
Source: Nature. 2021 Jun 16;594(7863):374–9. doi: 10.1038/s41586-021-03543-x (PMC8208892; doi:10.1038/s41586-021-03543-x)

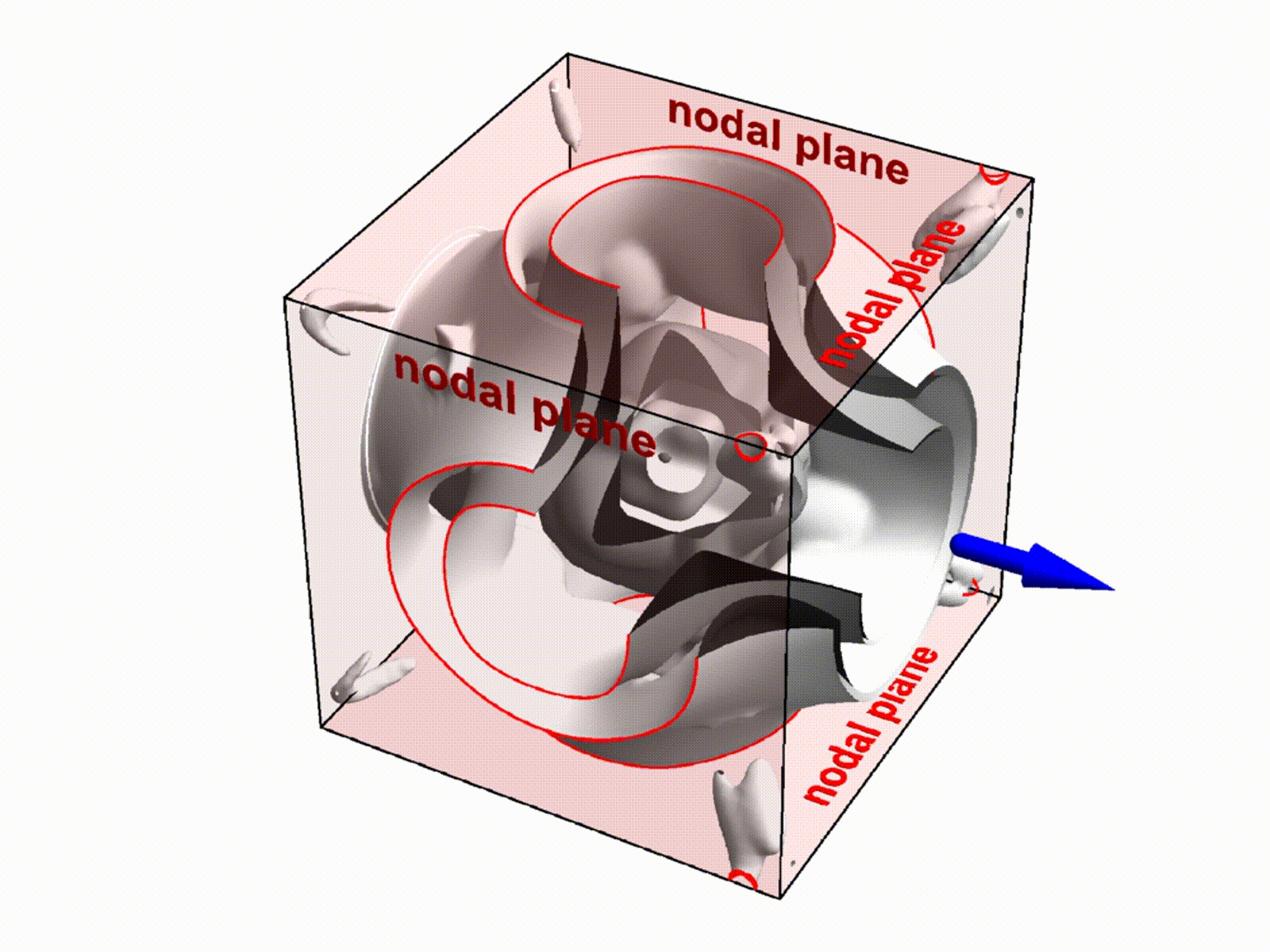

Supplement: Supplementary file 3 — This video highlights the evolution of a cut-away view of the Fermi surface akin Fig.1(f) as a function of the direction of the magnetization (blue arrow) following an applied magnetic field. Note the emergence of the topological degeneracies of Fermi surface pairs (5,6), (7,8) and (9,10) perpendicular to the direction of the magnetization. [file 41586_2021_3543_MOESM3_ESM.gif]
